# Association between neck circumference and diabetes mellitus: a systematic review and meta-analysis

**DOI:** 10.1186/s13098-023-01111-z

**Published:** 2023-06-21

**Authors:** Dandan Li, Yuxin Zhao, Lifang Zhang, Qiqi You, Qingqing Jiang, Xiaoxv Yin, Shiyi Cao

**Affiliations:** 1 Department of Medical Records Management, The First Affiliated Hospital of Zhengzhou University, Zhengzhou, Henan China; 2 Shenzhen Fuyong People’s Hospital, Shenzhen, Guangdong China; 3 Medical Service, The First Affiliated Hospital of Zhengzhou University, Zhengzhou, Henan China; 4 School of Public Health, Tongji Medical College, Huazhong University of Science and Technology, Wuhan, Hubei China

**Keywords:** Neck circumference, Type 2 diabetes mellitus, Gestational diabetes mellitus, Meta-analysis

## Abstract

**Background:**

Despite that several original researchers have investigated the association between neck circumference (NC) and the risk of diabetes mellitus (DM), their results remain controversial. This review aimed to quantitatively determine the risk of DM in relation to the NC.

**Methods:**

We conducted a literature search of PubMed, Embase, and the Web of Science from these databases’ inception through September 2022 to identify observational studies that examined the association between NC and the risk of DM. A meta-analysis of the random-effects model was applied to combine the results of the enrolled studies.

**Results:**

Sixteen observational studies involving 4,764 patients with DM and 26,159 participants were assessed. The pooled results revealed that NC was significantly associated with the risk of type 2 DM (T2DM) (OR = 2.17; 95% CI: 1.30–3.62) and gestational DM (GDM) (OR = 1.31; 95% CI: 1.17–1.48). Subgroup analysis revealed that after controlling for BMI, the relationship between the NC and T2DM remained statistically significant (OR = 1.94; 95% CI: 1.35–2.79). Moreover, the pooled OR of T2DM was found to be 1.16 (95% CI: 1.07–1.27) for an increment per each centimeter in the NC.

**Conclusions:**

Integrated epidemiological evidence supports the hypothesis that a greater NC is associated with an increased risk of T2DM and GDM.

**Supplementary Information:**

The online version contains supplementary material available at 10.1186/s13098-023-01111-z.

## Background

The incidence of diabetes mellitus (DM) has increased substantially in recent decades [1], which has imposed a heavy burden on healthcare systems. Knowledge of early markers to predict the disease and the adoption of related preventive strategies are of vital public health significance for improving this situation.

Obesity is a well-established risk factor for DM [2]. Several studies have suggested that total body obesity and abdominal obesity, which can be assessed based on body mass index (BMI), waist circumference (WC), and waist/hip ratio, could predict the risk of developing DM [3]. Recently, upper body subcutaneous fat has drawn the attention of researchers, and a study has shown that it may confer higher risks than visceral abdominal fat [4]. Furthermore, neck circumference (NC) has been considered as a proxy measure for upper body subcutaneous fat distribution [5]. Compared with other anthropometric indexes, NC measurement is more convenient, shows minimal fluctuations, and is not affected by respiratory conditions and diet. A meta-analysis [6] indicated that NC was moderately accurate in identifying overweight and obesity in children and adolescents, and another study [7] arrived at a similar conclusion in men and women of different age groups. Moreover, some studies have demonstrated that NC may be independently correlated with metabolic risk factors above and beyond BMI and WC [5, 8].

Over the past decades, numerous studies have assessed the relationship between NC and DM, but the results remain inconsistent. Some investigations have reported that NC has a direct relationship with DM [9–11], whereas others have shown that larger NC is not associated with the risk of DM [12, 13]. Considering the inconsistencies in the findings of existing studies, a systematic review and meta-analysis of observational epidemiological studies were performed to evaluate the association between NC and the risk of DM.

## Methods

### Literature search strategy

The present systematic review and meta-analysis were conducted in accordance with the Preferred Reporting Items for Systematic Reviews and Meta-Analysis (PRISMA) statement [14]. PubMed, Embase, and the Web of Science were searched from their inception until September 2022 using the following keywords with no restrictions to identify the relevant citations: ‘neck circumference’ in combination with ‘diabetes,’ ‘impaired glucose tolerance,’ ‘impaired fasting glucose,’ or ‘insulin resistance.’ The reference lists of the retrieved articles were also reviewed to identify any other pertinent studies.

### Study selection

The studies were included in the meta-analysis if they met the following inclusion criteria: (i) the study design was cross-sectional, cohort or case-control, (ii) the odds ratio (OR) or relative risk (RR) with 95% confidence interval (CI) of DM incidence related to the NC were reported or could be calculated from the provided data. Abstracts, non-original papers (reviews, editorials, or letters), gray literature, unpublished studies, and studies providing data on the relationship between the NC and diabetes-led mortality or complications were excluded. If there was more than one report from the same study, we only included the report with the most detailed information for both NC and the outcome.

### Data extraction

Two authors (D.L. and Y.Z.) independently extracted the following information from the included studies: first author, publication year, country or region, study design, age, sample size, the number of men and women, the cutoff point for NC, adjusted OR/RR with 95% CI, and the adjusted factors. The differences in data extraction between the two investigators were resolved via discussion with the third investigator (S.C.).

### Quality assessment

We assessed the methodological quality of cohort and case-control studies with reference to the Newcastle–Ottawa Scale, which awards a score of 0–9 based on the selection of participants, comparability of the groups, and exposure/outcome assessment [15]. Studies scoring 0–3, 4–6, and 7–9 were categorized as low-, moderate-, and high-quality studies, respectively.

The assessment tool involving 11 items, as recommended by the Agency for Healthcare Research and Quality, was applied for cross-sectional studies [16]. The quality of the studies was first evaluated with reference to the established questions and then scored according to the following criteria: 1 point = if the item was considered in the study and 0 points = if the item was not considered or this aspect was ambiguous.

Each study was rated independently by two authors (D.L. and Y.Z.). Any disagreements were resolved via discussion with a third investigator (S.C.).

### Statistical analysis

We considered OR as the common measure of association between the NC and the risk for DM. The reported RR was considered approximately as the OR. We calculated an overall pooled OR by using a random-effects model for the main analysis [17]. If studies reported results separately for different subgroups, we combined the subgroup estimates by using fixed-effect models before inclusion in the main meta-analysis. *Q* statistic with a significance level of < 0.10 and *I*^*2*^ statistic were applied to test the heterogeneity. The *I*^*2*^ statistic measures the percentage of total variation across studies because of heterogeneity rather than because of chance. It was calculated according to the formula by Higgins [18]. Substantial heterogeneity is an *I*^*2*^ value of at least 50%. Sensitivity analyses and subgroup analyses were conducted to evaluate the influences of the study design and population characteristics on our results. All statistical analyses were conducted by using STATA statistical software version 13.0 (STATA Corp, College Station, Texas, USA). *P* values were two-sided with a significance level of 0.05.

## Results

### Study selection and evaluation

**Fig. 1:**
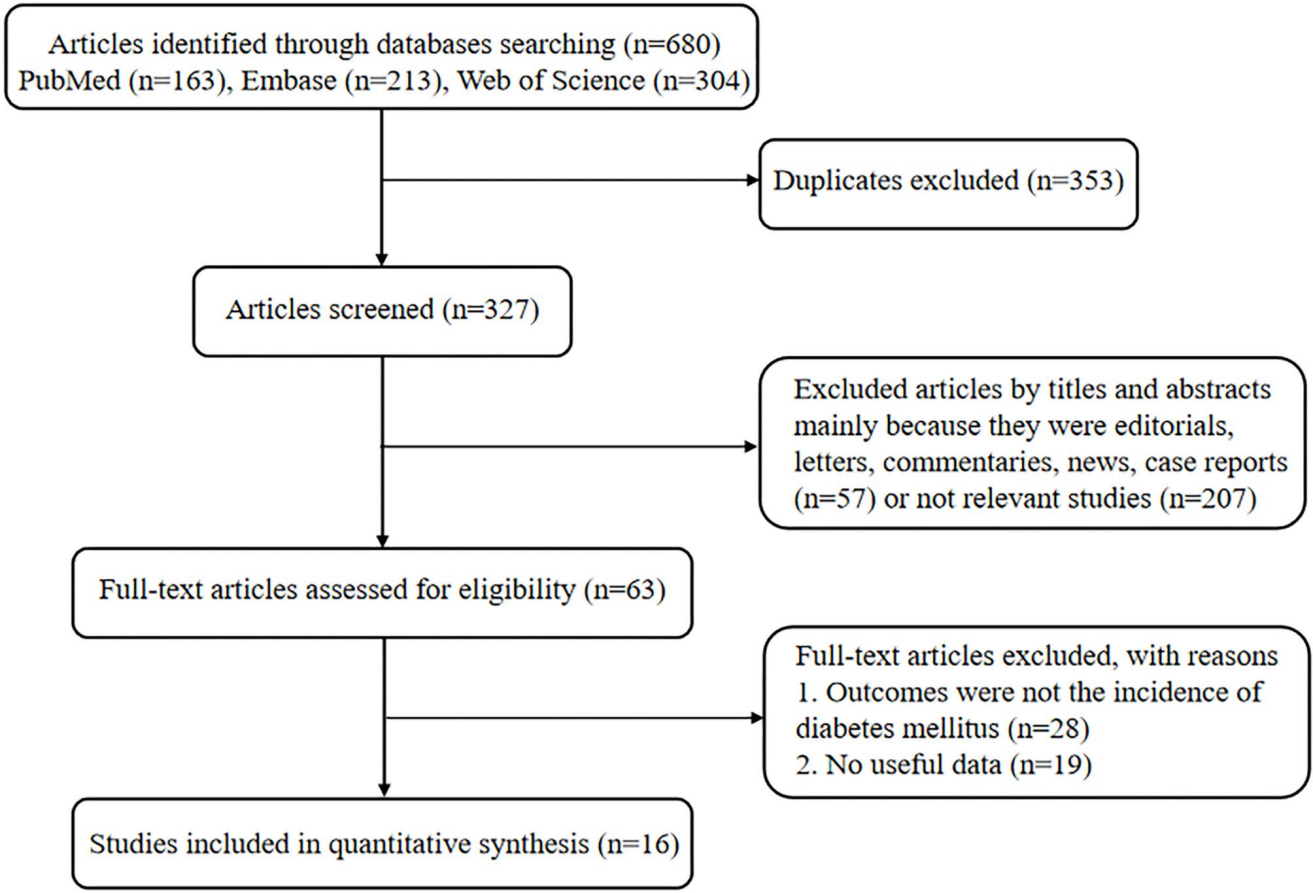
Flow diagram of studies included in the systematic review and meta-analysis

The results of the literature research and selection are illustrated in Fig. 1. Initially, we retrieved 163 citations from PubMed, 213 from Embase, and 304 from the Web of Science. After 353 duplicates were excluded, 327 citations were screened through titles and abstracts, of which 264 were excluded because they were reviews, editorials, letters, commentaries, news reports, case reports, or irrelevant studies. After assessing the full texts of the remaining 63 articles, we excluded 47 articles, as 28 of these did not study the relationship between NC and DM incidence while 19 did not provide useful data to calculate these parameters. Finally, 16 studies [4, 9–13, 19–28] were included (including seven cohort studies, one case-control study, and eight cross-sectional studies).

### Study characteristics

The main characteristics of the 16 studies are summarized in Table 1. These studies were published between 2010 and 2022. The study samples ranged in size from 196 to 8,450, with a total of 26,159, while the number of cases of DM ranged from 29 to 2,068, with a total of 4,764. Of these studies on the relationship between NC and DM, nine were about type 2 DM (T2DM), and seven were about gestational DM (GDM). None of the studies focused on the risk of other types of diabetes in relation to the NC. The study locations were as follows: nine studies were from Asia, three from Europe, three from South America, and one from North America. In addition, the quality scores for the seven cohort studies ranged from 5 to 9, with an average score of 7.43 from a maximum of 9 (Table A.1). The quality score for one case-control study was 7 (Table A.2). The quality assessment scores for the eight cross-sectional studies ranged from 4 to 7, with a mean score of 5 from a maximum of 11 (Table A.3).

**Table 1:** Main characteristics of the included studies involving neck circumference and the risk of diabetes mellitus

Author	Year	Disease	Country (state)	Study design	Age (years)	Simple size				Cutoff point (cm)	Adjustment
						Total	Case	Male	Female		
Sarah Rosner Preis et al.	2010	T2DM	USA (North America)	Cross-sectional	49.8 ± 10.7 (M) 52.1 ± 9.9 (F)	3307	193	1718	1589	NR	gender, BMI, waist, age, smoking, alcohol, menopausal status, and hormone replacement therapy use
Nam H. Cho et al.	2015	T2DM	Korea (Asia)	Cohort	49.8 ± 7.1(M) 50.6 ± 7.6 (F)	2623	632	NR	NR	NR	age, gender, BMI, family history of DM
Mykolay Khalangot et al.	2016	T2DM	Ukraine (Europe)	Cross-sectional	≥ 44	196	54	46	150	38.5 (M) 36.5 (F)	gender, BMI
Aline Marcadenti et al.	2017	T2DM	Brazil (South America)	Cross-sectional	18–80	430	142	145	285	NR	age, physical activity, smoking and BMI
Yavor Assyov et al.	2017	T2DM	Bulgaria (Europe)	Cross-sectional	49 ± 12	255	29	102	153	38 (M) 35 (F)	gender
Mingkuo Ting et al.	2018	T2DM	China (Asia)	Cohort	51.1 ± 11.9	8450	2068	4431	4019	NR	age, sex, education, marital status, occupation, betel nut chewing, and hypertension history, waist width, left thigh circumference, right upper arm circumference
Aléxei Volaco et al.	2018	T2DM	Brazil (South America)	Cross-sectional	46.5 ± 18.6 (M) 47.4 ± 17.6 (F)	950	170	329	621	39.5 (M) 34.5 (F)	gender, education, race, smoking, excessive alcohol consumption
Wenning Fu et al.	2019	T2DM	China (Asia)	Cross-sectional	56.0 ± 9.8	4000	387	1605	2395	36.6 (M) 33.40 (F)	age, sex, smoking, drinking and education
Qun Yan et al.	2021	T2DM	China (Asia)	Cohort	71.0 ± 5.8	2646	219	1288	1358	NR	age, exercise, current smoking, alcohol use, BMI, waist circumference, systolic blood pressure, diastolic blood pressure, triglycerides, total cholesterol, alanine aminotransferase
Fang He et al.	2017	GDM	China (Asia)	Case-control	29.1 ± 3.7	255	41	NA	255	NR	hemoglobin A1c, 1-h glucose, 2-h glucose, waist circumference, fasting blood glucose
Ping Li et al.	2018	GDM	China (Asia)	Cross-sectional	30 (27–32)	371	97	NA	371	33.8	BMI, maternal age, gravidity and parity
Lilian C Mendoza et al.	2018	GDM	Spain, Austria, Belgium, Denmark, Poland, Italy, Ireland, The Netherlands, The United Kingdom (Europe)	Cross-sectional	32.1 ± 5.3	971	425	NA	971	NR	maternal age, ethnicity, education, marital status, working status, obstetric history, BMI
Necati Hancerliogullari et al.	2020	GDM	Turkey (Asia)	Cohort	31 (19–41) (GDM); 27 (18–44) (control)	525	49	NA	525	38.5	age, gravidity, parity, BMI
Tahoora Sedighi Barforoush et al.	2021	GDM	Iran (Asia)	Cohort	28.1 ± 4.4	372	74	NA	372	34.3	age, BMI, fasting blood glucose
Azam Ghorbani er al.	2022	GDM	Iran (Asia)	Cohort	29.78 ± 4.91	676	110	NA	676	33.5	age, gravid, BMI, waist circumference
Camila Rodrigues de Souza Carvalho et al.	2022	GDM	Brazil (South America)	Cohort	32(6) (GDM); 28(6) (without GDM)	372	74	NA	372	34.5	age, physical activity, education, and familiar history of diabetes

Abbreviations: T2DM, type 2 diabetes mellitus; GDM, gestational diabetes mellitus; M, male; F, female; BMI, body mass index; NR, not reported; NA, not applicable

### Results of the meta-analysis

#### Association between the NC and T2DM

**Fig. 2:**
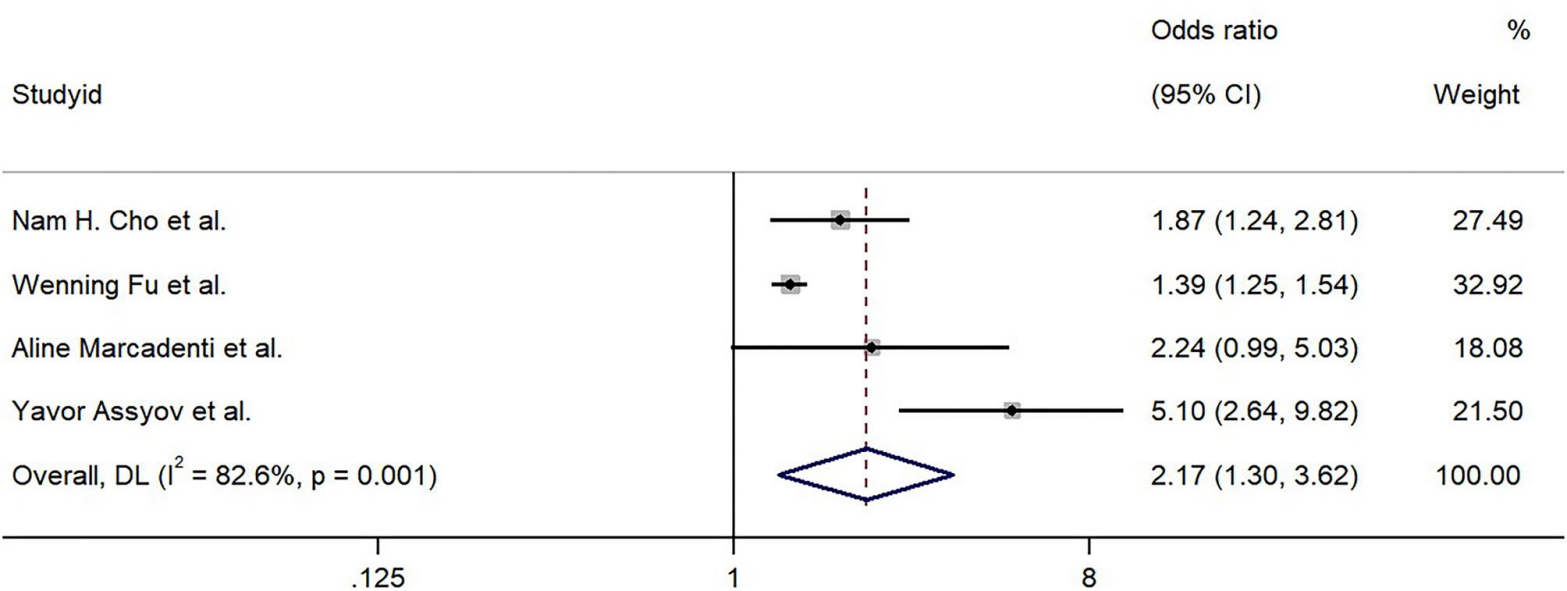
The association between neck circumference as a categorical variable and type 2 diabetes mellitus risk

The results from the random-effects model combining the ORs for the relationship of T2DM with NC are shown in Fig. 2. When compared with people with smaller NC, those with larger NC were at an increased risk for T2DM, and the pooled OR was 2.17 (95% CI: 1.30–3.62). Substantial heterogeneity was observed (*I*^*2*^ = 82.6%, *P =* 0.001).

**Fig. 3:**
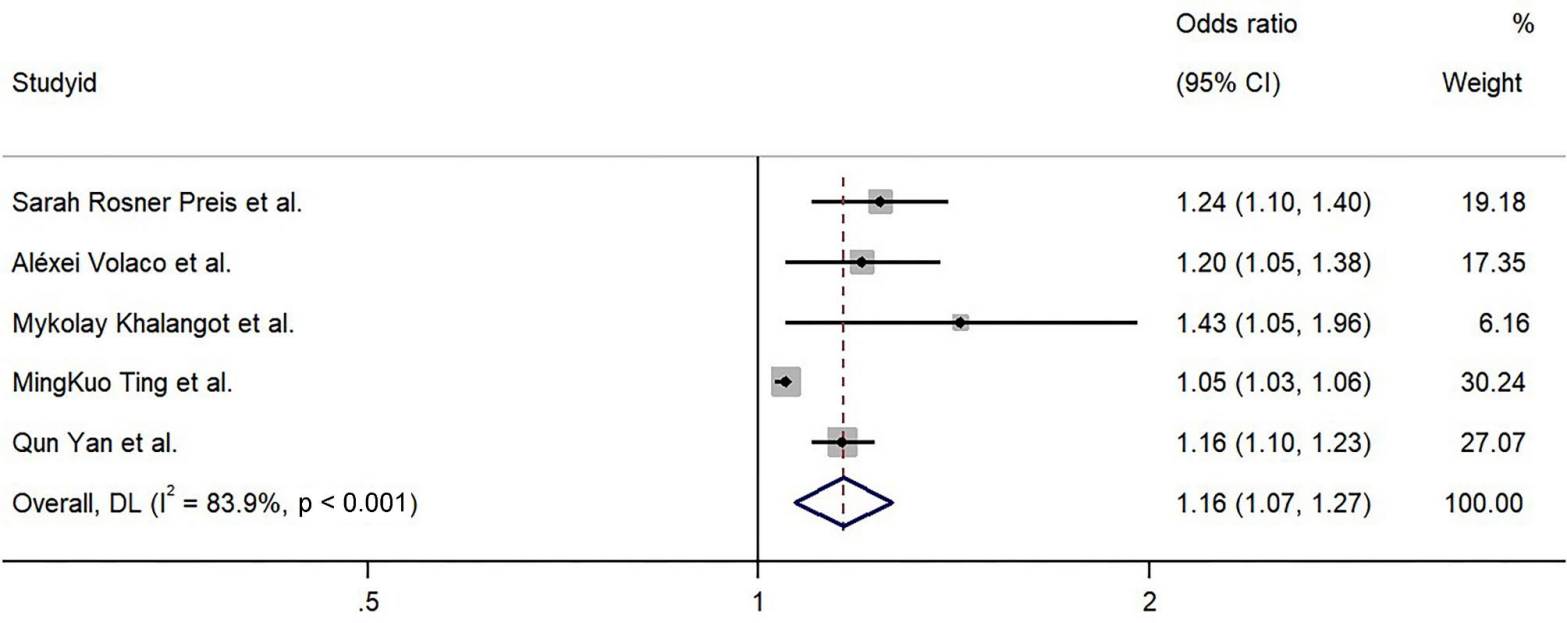
The association between neck circumference as a continuous variable and type 2 diabetes mellitus risk

Two studies [4, 19] evaluated the risk of T2DM for per standard deviation increment in the NC, and the OR with 95% CI was 1.70 (95% CI: 1.35–2.13) and 2.06 (95% CI: 1.71–2.49), respectively. Three other studies [11, 21, 26] reported the risk of T2DM per 1-cm increase in the NC, and the OR with 95% CI was 1.43 (95% CI: 1.05–1.96), 1.05 (95% CI: 1.03–1.96), and 1.16 (95% CI: 1.10–1.23), respectively. We standardized the two results reporting OR per standard deviation increment in the NC to the form of OR per 1-cm increment and calculated the pooled OR by using a random-effects model. The pooled OR of T2DM for an increment per each centimeter in the NC was 1.16 (95% CI: 1.07–1.27), with substantial heterogeneity across studies (*I*^*2*^ = 83.9%, *P* < 0.001) (Fig. 3).

##### Results of sensitivity analyses and subgroup analyses

To identify the potential influence of a single study on the pooled results, any single study was excluded in turn and the results of the remaining included studies were pooled. The pooled OR did not materially change and ranged from 1.55 (95% CI: 1.22–1.99) to 2.71 (95% CI: 1.43–5.14) (Fig. A1).

Table A.4 shows the results of subgroup analyses on the NC and T2DM risk. A larger NC was found to be associated with an increased risk for T2DM in most subgroups. Subgroup analysis by the state revealed that the participants from South America and Europe had a higher risk of T2DM, and the highest point estimate was recorded for Europe (OR = 5.10, 95% CI: 2.64–9.82). The difference in the pooled OR among these three groups showed statistical significance (*P* = 0.003). Subgroup analysis by controlling for age also indicated a statistically significant difference in results (*P =* 0.001). No significant difference was detected between the groups in terms of other variables. Notably, subgroup analyses by controlling for BMI indicated that the heterogeneity mainly arose from studies with unadjusted BMI (*I*^*2*^ = 93.2% for studies with unadjusted BMI and *I*^*2*^ = 0% for studies with adjusted BMI).

#### Association between the NC and GDM

**Fig. 4:**
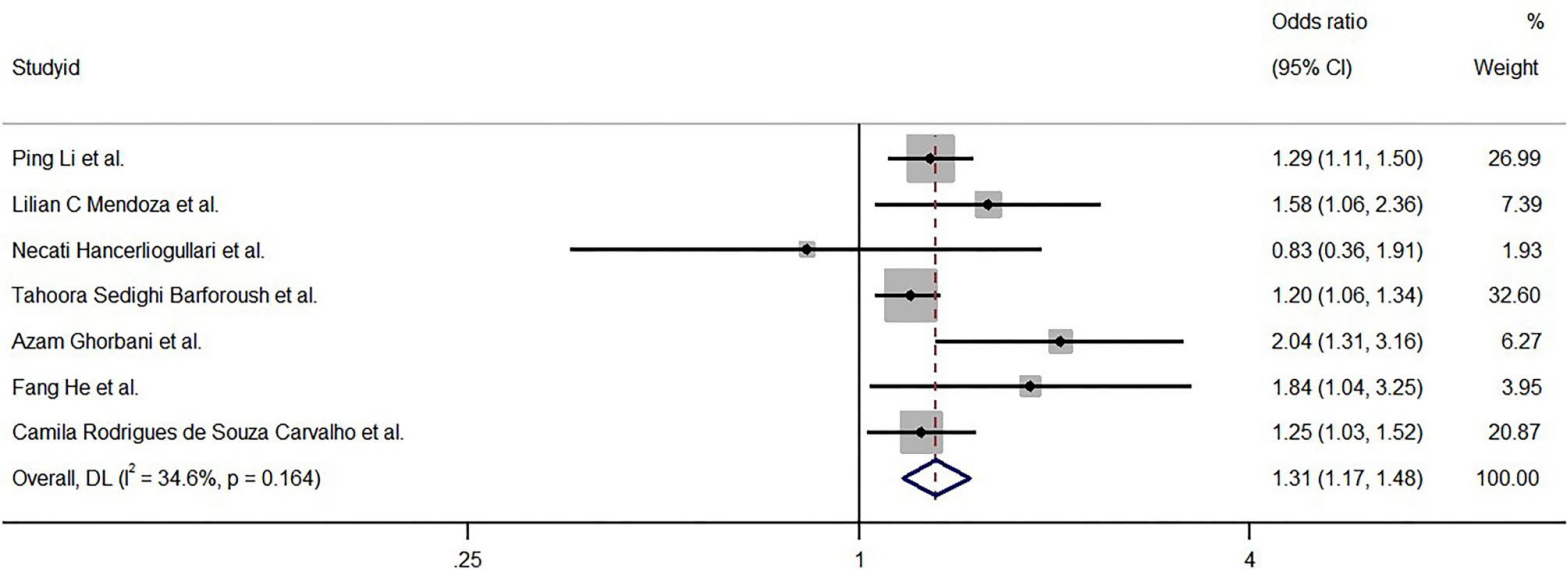
The association between neck circumference as a categorical variable and gestational diabetes mellitus risk

The results from the random-effects model combining the ORs for the relationship between GDM and NC are depicted in Fig. 4. The pooled OR was 1.31 (95% CI: 1.17–1.48), and no significant heterogeneity was observed (*I*^*2*^ = 34.6%, *P* = 0.164).

##### Results of sensitivity analyses and subgroup analyses

The sensitivity analysis indicated that the results were unaffected by any single study, with the pooled OR ranging from 1.25 (95% CI: 1.16–1.40) to 1.36 (95% CI: 1.15–1.61) (Fig. A2).

Table A.5 demonstrates the results of subgroup analyses about the NC and GDM risk. Subgroup analyses by the study design, state, and the cut-off point for NC, whether controlling for age and controlling for BMI showed no statistically significant difference in the outcomes. Most subgroups indicated a positive and statistically significant relationship between the NC and an increased risk of GDM.

## Discussion

NC, a novel anthropometric index, is considered a marker of subcutaneous fat distribution in the upper body and an independent predictor of metabolic disorders, such as glucose intolerance, hypertension, and fatty liver disease [29–31]. This systematic review and meta-analysis focused on the link between NC and the risk of DM. The investigation included 16 observational epidemiological studies involving 4,764 patients with DM and 26,159 participants. Pooled analysis revealed that NC was positively associated with the risk of DM. Specifically, compared with individuals who had smaller NC, those with larger NC had a 2.17 times increased risk of T2DM. Moreover, compared with pregnant women who had smaller NC, the risk of GDM was increased by 31% for those with larger NC.

Several potential mechanisms have so far been proposed to describe the relationship between NC and DM. First, NC is correlated positively with triglyceride levels and negatively with high-density lipoprotein cholesterol levels, both of which are robust markers for decreased insulin sensitivity [9, 32]. Additionally, larger NC with enhanced sympathetic activity may contribute to insulin resistance, thereby resulting in the development of DM [9]. Second, high NC values serve as a predictor of obstructive sleep apnea in short-sleeping obese men and women [33]. Certain studies have documented that obstructive sleep apnea is related to abnormal glucose metabolism [34, 35].

Owing to the substantial heterogeneity in studies exploring the association between NC and the risk of T2DM, subgroup analyses were conducted based on various factors. The findings showed that the association between NC and T2DM risk remained significant in most subgroups. The percentage and distribution of body fat for the same BMI varies across different populations [36]. Therefore, a subgroup analysis based on the state was performed, which revealed significant differences. Considering the potential differences between men and women, sex-based subgroup analyses were conducted, the results of which demonstrated that larger NC was a risk factor for T2DM in both sexes. Moreover, subgroup analyses based on adjusted variables, such as BMI and age, were performed to explore their possible influence on the relationship between NC and T2DM risk. According to the obtained results, after adjusting for age, the combined OR was lower than the unadjusted one. The difference between the two groups was statistically significant, which indicated that age was a positive confounder and that the true correlation effect between NC and T2DM may be weaker.

NC exhibits several advantages against previously used indices, such as BMI and WC. Although BMI is the most widely used index for defining overweight and obesity, it cannot assess body fat distribution. Likewise, although WC is a commonly used index for evaluating abdominal obesity, it fluctuates greatly and can be easily affected by conditions and time [37]. NC is stable, time-saving, and convenient to measure. Previous studies have observed that NC performs better than WC in evaluating metabolic health [38] and that it can predict excess body fat [39] and cardiovascular risk factors [40]. This may suggest that NC can be considered in guidelines for assessing obesity, especially when conventional anthropometric measures are not available, convenient, or practicable [38].

Our study has several strengths. First, in this systematic review and meta-analysis, the relationship between NC and T2DM was evaluated for the first time. Although Rahnemaei FA et al.’s study involved NC and DM, the researchers concentrated on the relationship between various anthropometric indicators and GDM [41]. Second, consistent results from sensitivity analyses among the included studies indicate the robustness and reliability of our findings.

However, there are some limitations, which are of concern. First, adjusted confounders varied among the included studies. Some probably important residual confounders, such as BMI, sex, and age, were not adjusted in certain studies. Second, different cutoff points for NC size were defined across studies, which might have introduced heterogeneity in the obtained results. Third, publication bias was not evaluated owing to the small number of studies on T2DM risk and GDM risk that were included in the meta-analysis in relation to the NC.

## Conclusion

The findings from this meta-analysis suggests that the risk of T2DM is elevated in individuals with a high NC. Moreover, pregnant women with high NC values have higher odds of GDM than those with low values. Nonetheless, the number of included studies was limited, and some possibly important residual confounders, such as BMI, were not adjusted in certain studies. Thus, more high-quality studies are required to confirm the predictive potential of NC for DM.

## Electronic supplementary material

Below is the link to the electronic supplementary material.

Supplementary Material 1

## Data Availability

The datasets used during the current study are available from the corresponding author on reasonable request.

## Abbreviations

BMI: Body mass index
CI: Confidence interval
DM: Diabetes mellitus
GDM: Gestational diabetes mellitus
NC: Neck circumference
OR: Odds ratio
RR: Relative risk
T2DM: Type 2 diabetes mellitus
WC: Waist circumference
