# Supplementary material for: Association between neck circumference and diabetes mellitus: a systematic review and meta-analysis
Source: Diabetol Metab Syndr. 2023 Jun 21;15:133. doi: 10.1186/s13098-023-01111-z (PMC10283198; doi:10.1186/s13098-023-01111-z)
Supplement: Supplementary file 1 — Supplementary Material 1 [file 13098_2023_1111_MOESM1_ESM.docx]

**Additional file 1**

**Table A.1** The quality assessment results of included cohort studies on neck circumference in relation to diabetes mellitus.

| Study | Selection | | | | Comparability | Outcome | | | Overall  quality  score |
| --- | --- | --- | --- | --- | --- | --- | --- | --- | --- |
|  | Exposed  group  represents  average in  community | The  comparison  groups  from the  same  source  population | Ascertain  exposure  through  records or  structured  interview | Demonstration  that outcome  not present at  study start | Comparability of cohorts on the basis of the design or analysis  (There was adequate  adjustment for  confounding in the  analyses from which  the main findings  were drawn) | Ascertain  outcome via  independent  blind  assessment  or medical  records,  physicians | Follow-up  long  enough for  outcome  to occur | Loss to  follow-up  <20% |  |
| Nam H. Cho et al. 2015 | 1 | 1 | 1 | 1 | 2 | 1 | 1 | 0 | 8 |
| Mingkuo Ting et al. 2018 | 1 | 1 | 1 | 0 | 1 | 1 | 1 | 0 | 6 |
| Necati Hancerliogullari et al. 2020 | 1 | 1 | 1 | 1 | 1 | 1 | 1 | 1 | 8 |
| Qun Yan et al. 2020 | 1 | 1 | 1 | 1 | 2 | 1 | 1 | 1 | 9 |
| Tahoora Sedighi Barforoush  et al. 2021 | 1 | 1 | 1 | 1 | 1 | 1 | 1 | 1 | 8 |
| Azam Ghorbani et al. 2022 | 1 | 1 | 1 | 1 | 1 | 1 | 1 | 1 | 8 |
| Camila Rodrigues de Souza Carvalho et al. 2022 | 1 | 1 | 1 | 0 | 1 | 1 | 0 | 0 | 5 |

The study quality was assessed according to the Newcastle-Ottawa quality assessment scale for cohort studies. This scale awards a maximum of 9 points to each study: 4 for selection, 2 for comparability, and 3 for assessment of outcomes (for cohort study). 1 = “Yes”, 0 = “No”, “Unable to determine” or “Not available”.

**Table A.2** The quality assessment results of included case-control studies on neck circumference in relation to diabetes mellitus.

| Study | Selection | | | | Comparability | Exposure | | | Overall  quality  score |
| --- | --- | --- | --- | --- | --- | --- | --- | --- | --- |
|  | The case definition with independent validation | Representativeness of the cases (consecutive or obviously representative series of cases) | Control group is selected from community | The definition of controls with no history of disease (endpoint) | Comparability of cases and controls on the basis of the design or analysis (There was adequate  adjustment for  confounding in the  analyses from which  the main findings  were drawn) | Ascertain exposure via  medical  records or structured interview where blind to case/control status | Same method of ascertainment for cases and controls | Same non-response rate for both groups |  |
| Fang He et al. 2017 | 1 | 1 | 0 | 1 | 2 | 1 | 1 | 0 | 7 |

The study quality was assessed according to the Newcastle-Ottawa quality assessment scale for case-control studies. This scale awards a maximum of 9 points to each study: 4 for selection, 2 for comparability, and 3 for assessment of exposures (for case-control study). 1 = “Yes”, 0 = “No”, “Unable to determine” or “Not available”.

**Table A.3** The quality assessment results of included cross-sectional studies on neck circumference in relation to diabetes mellitus.

| Study | 1.Define the source  of information | 2. List inclusion and exclusion criteria for exposed and unexposed subjects or refer to previous publications | 3. Indicate time period  used for identifying  patients | 4. Indicate whether or not subjects were consecutive if not population-based | 5. Indicate if evaluators of subjective components of study were masked to other aspects of the status of the participants | 6. Describe any assessments undertaken for quality assurance purposes | 7. Explain any patient exclusions from analysis | 8. Describe how confounding  was assessed and/or controlled | 9. If applicable, explain how missing data were handled in the analysis | 10. Summ-arize patient response rates and completeness of data collection | 11. Clarify what follow-up, if any, was expected and the percentage of patients for which incomplete data or follow-up was obtained | Overall  quality  score |
| --- | --- | --- | --- | --- | --- | --- | --- | --- | --- | --- | --- | --- |
| Sarah Rosner Preis et al. 2010 | 1 | 0 | 1 | 1 | 0 | 0 | 1 | 1 | 1 | 0 | 1 | 7 |
| Mykolay Khalangot et al. 2016 | 1 | 0 | 1 | 1 | 0 | 0 | 0 | 1 | 0 | 1 | 0 | 5 |
| Aléxei Volaco et al. 2017 | 1 | 1 | 1 | 1 | 0 | 0 | 1 | 0 | 0 | 0 | 0 | 5 |
| Yavor Assyov et al. 2017 | 1 | 1 | 1 | 1 | 0 | 0 | 0 | 0 | 0 | 0 | 0 | 4 |
| Wenning Fu et al. 2019 | 1 | 1 | 1 | 1 | 0 | 0 | 1 | 1 | 0 | 0 | 0 | 6 |
| Aline Marcadenti et al. 2017 | 1 | 1 | 0 | 1 | 0 | 0 | 0 | 1 | 0 | 0 | 0 | 4 |
| Ping Li et al. 2018 | 1 | 1 | 1 | 1 | 0 | 0 | 0 | 1 | 0 | 0 | 0 | 5 |
| Lilian C Mendoza et al. 2018 | 1 | 1 | 0 | 1 | 0 | 0 | 0 | 1 | 0 | 0 | 0 | 4 |

The study quality was assessed according to the 11 items recommended by the Agency for Healthcare Research and Quality (AHRQ) for cross-sectional studies. 1 point if the item was contemplated in the study, 0 point if the item was not and unable to determine. 1 = “Yes”, 0 = “No”, “Unable to determine”, or “Not applicable”.

**Table A.4** Results of subgroup analyses about neck circumference and type 2 diabetes mellitus risk.

| **Subgroup** | **Number of studies** | **OR/RR** | **95% confidence intervals** | ***P* for heterogeneity** | ***I^2^*** | ***P* value between groups** |
| --- | --- | --- | --- | --- | --- | --- |
| Study design |  |  |  |  |  |  |
| Cohort | 1 | 1.87 | 1.24–2.81 | - | - | 0.596 |
| Cross | 3 | 2.42 | 1.03–5.68 | < 0.001 | 87.4% |  |
| State |  |  |  |  |  |  |
| Asia | 2 | 1.51 | 1.16–1.95 | 0.168 | 47.3% | 0.003 |
| South America | 1 | 2.24 | 0.99–5.03 | - | - |  |
| Europe | 1 | 5.10 | 2.64–9.82 | - | - |  |
| Gender |  |  |  |  |  |  |
| Male | 4 | 1.85 | 1.17–2.91 | 0.032 | 66.0% | 0.534 |
| Female | 4 | 2.33 | 1.30–4.18 | 0.002 | 79.2% |  |
| Controlling for age |  |  |  |  |  |  |
| Yes | 3 | 1.55 | 1.22–1.99 | 0.212 | 35.6% | 0.001 |
| No | 1 | 5.10 | 2.64–9.84 | - | - |  |
| Controlling for BMI |  |  |  |  |  |  |
| Yes | 2 | 1.94 | 1.35–2.79 | 0.697 | 0 | 0.684 |
| No | 2 | 2.55 | 0.72–9.10 | < 0.001 | 93.2% |  |

Abbreviations: OR, odds ratio; RR, relative risk; BMI, body mass index.

**Table A.5** Results of subgroup analyses about neck circumference and gestational diabetes mellitus risk.

| **Subgroup** | **Number of studies** | **OR/RR** | **95% confidence intervals** | ***P* for heterogeneity** | ***I^2^*** | ***P* value between groups** |
| --- | --- | --- | --- | --- | --- | --- |
| Study design |  |  |  |  |  |  |
| Cohort | 4 | 1.29 | 1.07–1.56 | 0.107 | 50.9% | 0.975 |
| Case–control | 1 | 1.84 | 1.04–3.25 | - | - |  |
| Cross | 2 | 1.32 | 1.15–1.52 | 0.353 | 0.0% |  |
| State |  |  |  |  |  |  |
| Asia | 5 | 1.33 | 1.12–1.58 | 0.091 | 50.0% | 0.164 |
| South America | 1 | 1.25 | 1.03–1.52 | - | - |  |
| Europe | 1 | 1.58 | 1.06–2.36 | - | - |  |
| Cut-off point for NC ^a^ |  |  |  |  |  |  |
| < 35 cm | 4 | 1.28 | 1.14–1.45 | 0.147 | 44.1 | 0.310 |
| ≥ 35 cm | 1 | 0.83 | 0.36–1.91 | - | - |  |
| Controlling for age |  |  |  |  |  |  |
| Yes | 6 | 1.29 | 1.15–1.45 | 0.183 | 33.8% | 0.234 |
| No | 1 | 1.84 | 1.04–3.25 | - | - |  |
| Controlling for BMI |  |  |  |  |  |  |
| Yes | 5 | 1.33 | 1.13–1.55 | 0.110 | 46.9% | 0.839 |
| No | 2 | 1.38 | 0.99–1.91 | 0.209 | 36.8% |  |

Abbreviations: OR, odds ratio; RR, relative risk; NC, neck circumference; BMI, body mass index.

^a^ Two studies did not provide information on the cut-off point for NC.





Fig.A1 Sensitivity analysis by leave-one-out method about neck circumference and type 2 diabetes mellitus risk





Fig.A2 Sensitivity analysis by leave-one-out method about neck circumference and gestational diabetes mellitus risk
